# Immunotherapy-induced psoriasis successfully treated with Guselkumab in a patient with metastatic gastric cancer^☆^

**DOI:** 10.1016/j.abd.2024.08.009

**Published:** 2025-03-13

**Authors:** Denis Miyashiro, Thiago William Carnier Jorge, André Luís da Silva Hirayama, José Antonio Sanches

**Affiliations:** a Department of Dermatology, Faculty of Medicine, Universidade de São Paulo, São Paulo, SP, Brazil; b Dermatology Group, Hospital Alemão Oswaldo Cruz, São Paulo, SP, Brazil; c Department of Medical Oncology, Hospital Alemão Oswaldo Cruz, São Paulo, SP, Brazil

*Dear Editor,*

Programmed Death-1/Programmed Death Ligand-1 (PD-1/PD-L1) and Cytotoxic T-Lymphocyte Associated Protein-4 (CTLA-4) are regulatory proteins that inhibit T-cell activity.^1^ Immune checkpoint inhibitors remove the blockade of the immune system, allowing T-cells to act against malignant cells.^1^, ^2^ Due to the inactivation of these co-inhibitory receptors, immune-mediated adverse events may arise, affecting the gastrointestinal tract, lungs, endocrine system, kidneys, liver, eyes, musculoskeletal system, nervous system, and skin.^3^ Among cutaneous lesions associated with anti-PD-1 and anti-CTLA-4 drugs, maculopapular rash, psoriasis, pruritus, vitiligo, bullous pemphigoid, Stevens-Johnson syndrome/toxic epidermal necrolysis, and other less common disorders (drug reaction with eosinophilia and systemic symptoms, acute generalized exanthematous pustulosis, Sweet syndrome, pyoderma gangrenosum, dermatomyositis, vasculitis) were described.^4^ Immunotherapy-related psoriasis occurs *de novo* or as an exacerbation of previous psoriasis.^5^ We describe a case of immunotherapy-induced psoriasis in a patient with metastatic gastric cancer successfully treated with Guselkumab.

A 62-year-old male presented with plaques with moderate infiltration and mild scaling on the back, frontal, and pre-auricular regions for two months (Fig. 1). He had a previous diagnosis of stage IV gastric adenocarcinoma with liver metastasis and has been treated with nivolumab 240 mg every two weeks for six months with partial response. He had no history of previous cutaneous diseases and denied a family history of immune-mediated disorders. The hypothesis of nivolumab-induced psoriasis was raised. Skin biopsy revealed regular acanthosis, hypogranulosis, parakeratosis, dilated capillaries in the papillary dermis, and Munro’s microabscesses compatible with psoriasis (Fig. 2). Skin lesions were refractory to topical steroids and due to intense pruritus, the patient stopped cancer treatment. Because of the lack of response to topical steroids, difficulty in adhering to phototherapy, and contraindication for the use of acitretin and methotrexate due to liver metastasis, the patient started Guselkumab 100 mg at weeks 0-, 4-, and every 8-weeks. After two applications, significant improvement was observed (Fig. 3). After 12-months skin lesions completely resolved, but malignant disease progressed with new liver lesions. Nivolumab was restarted, and skin remains clear with no significant adverse events due to Guselkumab.

**Fig. 1:**
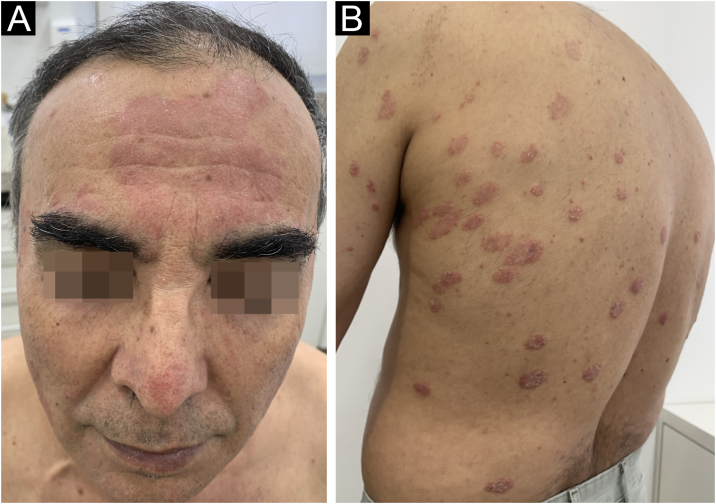
Erythematous and scaling plaques on the forehead (A), and back (B).

**Fig. 2:**
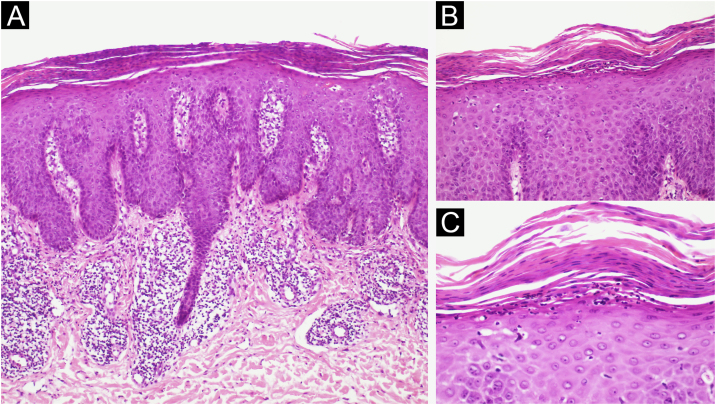
(A) Regular acanthosis, hypogranulosis, parakeratosis, dilated capillaries in the papillary dermis (Hematoxylin & eosin, ×100). (B) Parakeratosis and Munro’s microabscesses (B, Hematoxylin & eosin, ×200). (C) Higher magnification showing parakeratosis and Munro’s microabscesses (C, Hematoxylin & eosin, ×400).

**Fig. 3:**
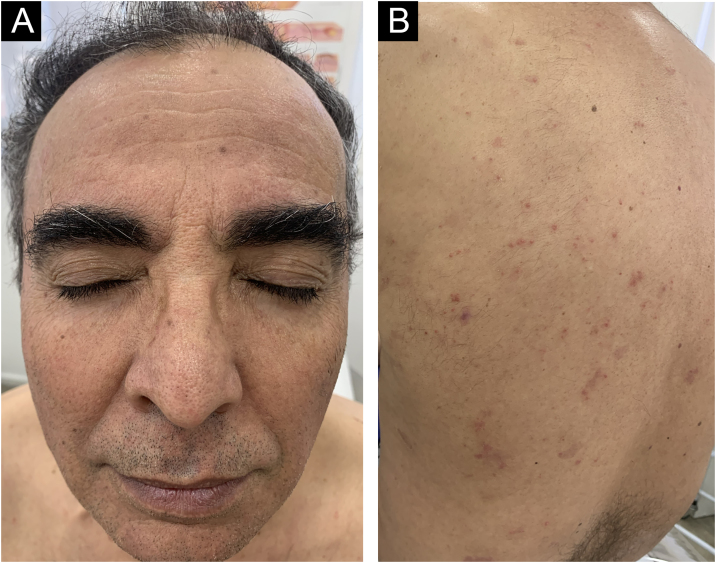
Significant improvement of skin lesions after 2 doses of Guselkumab.

Psoriasis associated with immune checkpoint inhibitors occurs in approximately 0.5% of patients.^6^ It may occur *de novo* (70%) or as an exacerbation of previous psoriasis (30%), and skin lesions usually appear 5‒12-weeks after the start of immunotherapy.^5^

Immune checkpoint inhibitors increase T-cell response against tumor cells, and the increased release of pro-inflammatory T-helper 1 (Th1) and 17 (Th17) cytokines may result in exacerbation or induction of psoriasis.^7^

A large retrospective study analyzing 7008 patients who developed cutaneous immune-related adverse events secondary to anti-PD-1 or anti-PD-L1 for treatment of different malignant diseases showed a strong association between the development of cutaneous lesions, including psoriasis, and response to immune-checkpoint inhibitors.^8^ Some studies show that guttate lesions and psoriasis affecting >10% of the body surface area are associated with better immunotherapy responses, but pruritus is a negative predictor of response.^5^

Treatments reported for immunotherapy-induced psoriasis include topical steroids, calcipotriol, phototherapy, and systemic therapies. The most common systemic agent administered is acitretin since it does not have immunosuppressive effects.^5^ The impact of biologics on the treatment of psoriasis in cancer patients is not fully understood. Most evidence shows no increased risk of cancer in patients with psoriasis, rheumatoid arthritis, or inflammatory bowel disease treated with anti-tumor necrosis factor-alpha, anti-interleukin 17, anti-interleukin 12 and/or 23, or Janus-kinase inhibitors.^9^, ^10^

Guselkumab is a fully human monoclonal antibody that inhibits interleukin 23 by binding to its subunit p19. The use of Guselkumab in patients with previous diagnoses of cancer has been investigated in small case series.^9^, ^10^

We reported the case of *de novo* psoriasis secondary to immune-checkpoint inhibitor therapy that was successfully treated with Guselkumab with rapid response and no significant adverse events. We highlight the importance of treating immune-mediated adverse events secondary to immunotherapy since the use of this type of drug is increasing rapidly due to its efficacy in different types of cancers, and we must be aware to treat these patients to provide a good quality of life and prevent from discontinuing or reducing the dosage of cancer treatment.

## Authors’ contributions

Denis Miyashiro: The study concept and design; data collection, or analysis and interpretation of data; Writing of the manuscript or critical review of important intellectual content; Effective participation in the research guidance; Intellectual participation in the propaedeutic and/or therapeutic conduct of the studied cases; Critical review of the literature; Final approval of the final version of the manuscript.

Thiago William Carnier Jorge: Data collection, analysis, and interpretation of data; Intellectual participation in the propaedeutic and/or therapeutic conduct of the studied cases; Critical review of the literature; Final approval of the final version of the manuscript.

André Luís da Silva Hirayama: Data collection, analysis, and interpretation of data; Intellectual participation in the propaedeutic and/or therapeutic conduct of the studied cases; Critical review of the literature; Final approval of the final version of the manuscript.

José Antonio Sanches: The study concept and design; Effective participation in the research guidance; Final approval of the final version of the manuscript.

## Financial support

None declared.

## Conflicts of interest

None declared.
